# Supplementary material for: Mutations in PCYT2 disrupt etherlipid biosynthesis and cause a complex hereditary spastic paraplegia
Source: Brain. 2019 Oct 22;142(11):3382–97. doi: 10.1093/brain/awz291 (PMC6821184; doi:10.1093/brain/awz291)
Supplement: awz291_Supplementary_Data [file awz291_supplementary_data.zip › awz291-Suppl_data/Supplementary_Data4.zip!/Supplementary information 3 - fibroblast lipidomics.zip!/Fibroblast lipidomics/Controle vs Patient1/Statistical plots and data/DG species/Volcano plot_alternative.pdf]

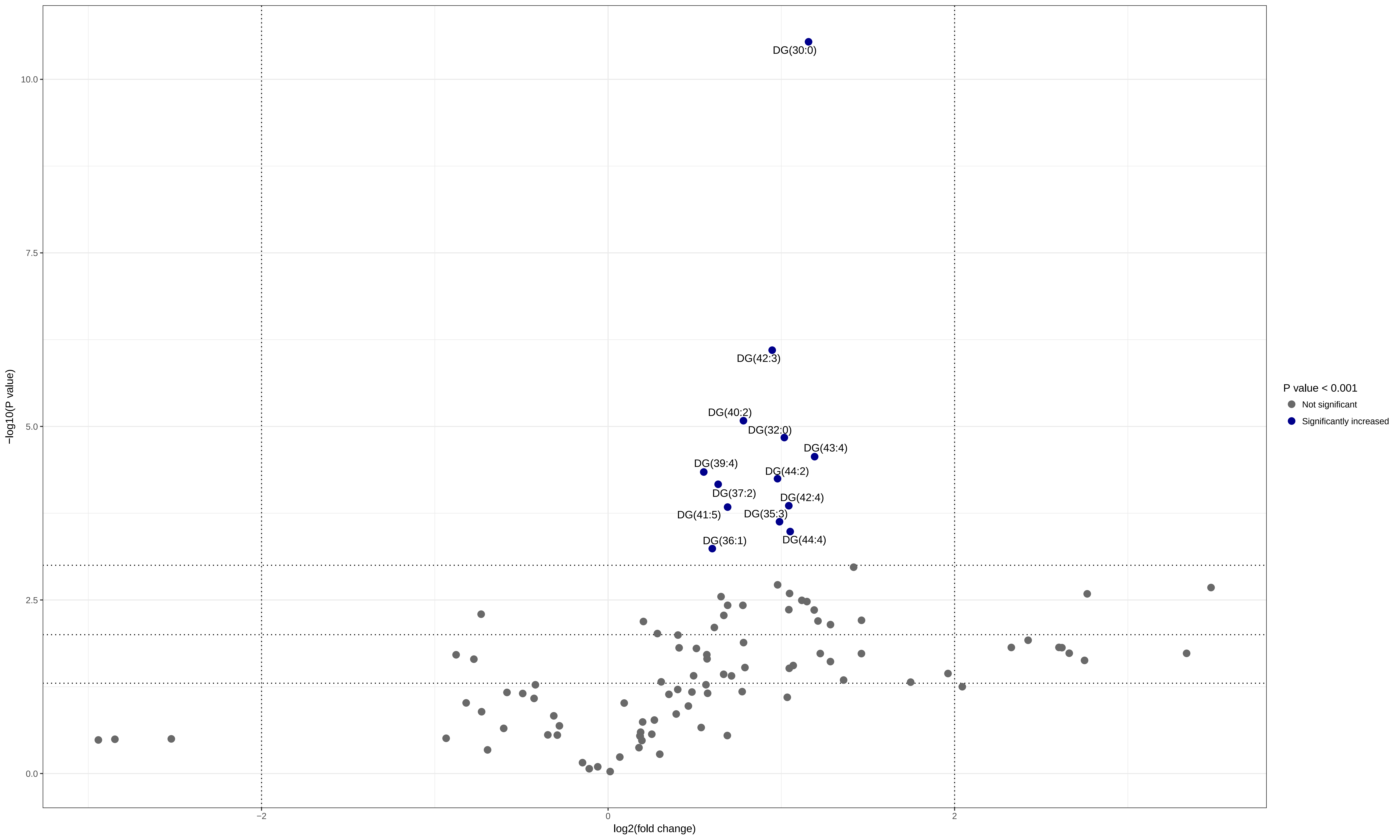

DG(30:0)
DG(42:3)
DG(40:2)
DG(32:0)
DG(43:4)
DG(39:4)
DG(44:2)
DG(37:2)
DG(42:4)
DG(41:5)
DG(35:3)
DG(44:4)
DG(36:1)
10.0
7.5
5.0
2.5
0.0
−2
0
2
−log10(P value)
log2(fold change)
P value < 0.001
Not significant
Significantly increased
